# The Association of Traumatic Brain Injury, post-traumatic stress disorder, and criminal recidivism

**DOI:** 10.1186/s40352-022-00169-7

**Published:** 2022-02-17

**Authors:** Pamela K. Lattimore, Nicholas J. Richardson, Pamela L. Ferguson, E. Elisabeth Pickelsimer

**Affiliations:** 1 RTI International, Research Triangle Park, North Carolina USA; 2 Medical University of South Carolina, South Carolina Charleston, USA

**Keywords:** Traumatic brain injury, TBI, Post-traumatic stress disorder, PTSD, Criminal recidivism, Violence, Prisoners

## Abstract

**Background:**

The purpose of the study was to assess the prevalence of traumatic brain injury (TBI) and post-traumatic stress disorder (PTSD) and to determine whether TBI or PTSD is associated with an increase in general or violent criminal recidivism among a representative sample of released prisoners.

In-person interviews were conducted with a stratified random sample of individuals incarcerated with the South Carolina Department of Corrections approximately 90 days prior to the prisoners’ releases. In addition to a variety of items and scales, respondents were screened for TBI and were asked whether they had received a current diagnosis of PTSD. Data were merged with arrest data that provided measures of past criminal involvement and indicators of post-release recidivism (arrest). Arrests were coded as “general” for any arrest charge and “violent” for any violent offense charge.

**Results:**

Survival analyses indicate that neither TBI nor PTSD predicts time to general recidivism. PTSD (*p* < 0.01) and age at first arrest (*p* < 0.01) are significant predictors for violent recidivism and TBI is non-significant at *p* = 0.09. Results from the negative binomial models indicate that TBI (*p* < 0.05) and PTSD (*p* < 0.05) are significantly associated with more post-release violent arrests, but not general arrests.

**Conclusions:**

TBI and PTSD were found to predict violent offending but not general criminal behavior. These findings demonstrate the need for prison officials to identify individuals with a history of TBI and PTSD and to develop appropriate interventions that could be provided during incarceration to reduce the post-release likelihood of violence.

## Introdu***c***tion

In recent years, traumatic brain injury (TBI) and post-traumatic stress disorder (PTSD) have been the subject of increasing scholarly attention, in part because of concerns about potential significant impacts on public health. It is estimated that more than 1.7 million people experience a TBI in the United States each year (Faul, et al., 2010). TBI is frequently referred to as the “silent epidemic” because many of the cognitive, behavioral, and social symptoms are not readily apparent (Fleminger, & Ponsford, 2005; Raine, et al., 1994; Williams, 2003). Although many individuals who experience TBI do not have long-term, persistent effects, some experience a range of social and psychological consequences including personality changes such as increases in irritability or aggressive behavior, poor impulse control, and a failure to plan ahead (Ferguson, et al., 2012). Without early identification and subsequent intervention, these individuals may pose a threat to themselves and others, including, perhaps, interactions with the criminal justice system. Although methodological issues flaw their findings, several researchers find that the prevalence of TBI among offenders is much higher than in the general population, with estimates ranging anywhere from 25% to 87% compared to approximately 8% in the general population (Barnfield, & Leathem, 1998; DelBello, et al., 1999; Langevin, 2006; Ray, & Sapp, 2014; Shiroma et al., 2012). These findings have resulted in additional research investigating how those with TBI differ from those without in their experiences with the criminal justice system.

Using a sample of male prisoners in the United Kingdom, Williams and colleagues (Williams, Mewse, et al., 2010) found that those with TBI were more likely to be incarcerated at a younger age. Additionally, those with TBI reported spending more time in custody over the previous five years than those without TBI and were also more likely to report histories of re-offending. In another study, Williams and his colleagues (Williams, Cordan, et al., 2010) examined the relationship between TBI in juvenile offenders and re-offending. The authors found that, among a sample of incarcerated juvenile offenders, the frequency of reported TBI was positively associated with the number of convictions.

PTSD affects approximately 6 to 9% of the general population and upwards of 20% of the incarcerated population and is characterized by re-experiencing a particular traumatic event, avoidance of the stimuli associated with the event, and hyperarousal (Cohen et al., 2003; Diagnostic and statistical manual of mental disorders, 2004; United States Department of Justice, 2013). These symptoms can often lead to a variety of feelings including severe helplessness and fear, while impeding normal day-to-day functioning (Cohen, et al., 2003; McFarlane, 2006). Because of the tremendous psycho-social impairment PTSD can have on individuals, many scholars have been investigating a possible link between PTSD and antisocial behavior, in particular, violence and aggression.

After a review of the literature, we identified several studies examining the relationship between PTSD, recidivism, and violence among criminal justice populations. Of particular salience to the current research, one study examined the effects of PTSD and substance use disorders on criminal recidivism using a sample of male and female incarcerated individuals (Goff, et al., 2007). The authors found that women experienced a greater number of traumatic events than men. Individuals with co-occurring trauma-related and substance use disorders have adverse post-incarceration outcomes (see Kubiak, 2004). Incarcerated individuals with PTSD were more likely to recidivate than inmates without a diagnosis of PTSD. Using a sample of over 4000 individuals recruited from a hospital in Atlanta, Georgia, Donley and colleagues examined the link between PTSD and involvement in the criminal justice system (Donley, et al., 2012). After controlling for a variety of known correlates of criminal behavior, the authors found that trauma exposure and PTSD are strongly associated with involvement in the criminal justice system.

Other studies have examined the relationship between PTSD and violent behavior, specifically. In a longitudinal sample of Finnish children born between 1986 and 2000, Peltonen and colleagues examined the relationship between early trauma on violent offending (Peltonen, et al., 2020). They found that even after controlling for known predictors of offending behavior including substance abuse and several mental health diagnoses, the findings indicated that trauma related disorders including PTSD were associated with subsequent violent offending. A meta-analysis of studies examining the association between PTSD and outcomes for prison populations, found a significant association between PTSD and violence and aggression among the incarcerated populationalthough most of the studies reflected cross-sectional designs that precluded identification of causal direction (Facer-Irwin, et al., 2019).

Other studies have examined the effects of PTSD in the context of soldiers returning home from combat. Much of this research focuses on Vietnam and Iraq war veterans for whom it is estimated that almost 30% of these individuals suffer from PTSD (McFall, et al., 1999). Findings from these studies suggest a positive link between PTSD and aggression and violent behavior (McFall, et al., 1999; Cicerone, et al., 2000; Whyte, et al., 1999; also see Freeman & Roca, 2001); Kristiansson et al., 2004; and Sherman et al., 2014). Additionally, those with PTSD were found to be more likely to abuse alcohol and drugs, which has been shown to lead to increases in violent behavior. From these findings, it is reasonable to hypothesize that those with PTSD may, at some point, engage in violent behavior that may lead to involvement with the criminal justice system.

These studies have several limitations. First, many scholars have focused on descriptive or cross-sectional studies comparing the rate of TBI or PTSD among offending populations to the rate of TBI or PTSD in the general population. Those types of research designs not only make it difficult to establish the causal ordering of variables, but they also fail to control for a variety of known predictors of criminal behavior. Second, although there have been a significant number of research studies on TBI or PTSD and general criminal behavior, few studies have examined prospectively whether TBI and PTSD are associated with criminal recidivism, and particularly violent recidivism. Lastly, no studies have included both TBI and PTSD in the same models. This is important as each has been shown to be associated with violence and criminal behavior and it is important to assess whether these key variables might interact, and if so, in what ways.

Thus, one understudied important research question is how individuals with TBI and PTSD fare post-incarceration. In other words, do those with TBI and PTSD have higher rates of criminal recidivism than those without? The goal of the current study is to fill in this gap by examining the relationship between PTSD and TBI and post-incarceration arrest. Our analysis provides a prospective, longitudinal study of a representative sample of inmates in South Carolina followed using administrative data for an average of nearly 700 days following release from prison. Additionally, approximately half the sample is female, which is important given that most previous studies only examine male populations. The analyses examine the impact of PTSD and TBI on the time to general and violent recidivism using survival analysis and the total numbers of post-release general and violent arrests using negative binomial regression. The analyses control for a variety of factors known to be associated with recidivism.

## Methods

Data were collected from a stratified random sample of individuals incarcerated by the SC Department of Correction (SCDC) during in-person interviews conducted by trained interviewers between April 2009 and April 2010 (Pickelsimer, et al., 2011; Ferguson, & Pickelsimer, 2011; Ferguson, et al., 2012). The purpose of the study was to assess the prevalence and frequency of PTSD and TBI among a random sample of incarcerated individuals and then determine any relationship between PTSD and TBI and post-release criminal outcomes (among those released). The interview data were supplemented with arrest data obtained from the South Carolina State Law Enforcement Division (SC SLED) in early 2013. These data provided an average of 692 days of post-release follow-up, with a minimum of 281 days and a maximum of 1324 days for those in the released sample.

Men and women were sampled separately every two months within three release strata: (1) “nonreleases,” long-term prisoners with life or death sentences; (2) parolees, individuals expected to be released on parole during the study period; and (3) “maxouts,” individuals expected to be released after serving their complete sentences some of whom were to be released to probation sentences) (Ferguson, et al., 2012). Individuals were excluded who appeared in a previous sample, were younger than 18 years of age, were non-English speaking, could not provide informed consent (e.g., due to intellectual disability or mental illness), were housed in special management units, were not arrested in SC or housed in a SCDC facility, or were expected to be released to law enforcement or to mental health facilities (the former indicating a detainer—warrants or holds lodged against an individual for possible future prosecution). Forty-one percent of selected individuals were not eligible, mostly due to detainers and repeated sample presence. In addition, individuals sentenced under the Youthful Offender Act (about 13% of individuals being released) could not be purposefully sampled because of indeterminate release dates. A total of 889 inmates were sampled to achieve our goal of 636 interviews. The 28% who were not interviewed either refused or were not available due to administrative issues (the latter usually due to the individual having been released before an interview could be scheduled and conducted).

Interviews were conducted with 294 men and 282 women expected to complete their sentences (although one male interviewee was not released during the data collection period) and 26 men and 34 women with either life or death sentences. Interviews with those expected to be released were conducted within 3 to 4 months prior to expected release. Because the purpose here is to examine post-release recidivism, only data for the 575 released inmates (293 men and 282 women) are included in the analyses.

Recidivism is defined here as rearrest in South Carolina following initial release. Arrests were coded as “violent” if the charge reflected a violent crime as defined by the National Corrections Reporting Program (United States Department of Justice,2013). “General” arrests are arrests for any offense including violent offenses. Specific recidivism measures were time to first violent arrest, time to first general arrest, counts of violent arrests, and counts of general arrests.

TBI was assessed using a modified version of the Ohio State University TBI Identification Method (OSU TBI-ID) (Bogner, & Corrigan, 2009; Corrigan, & Bogner, 2007). TBI was defined as having reported during the interview any external blow to the head with alteration of consciousness. PTSD was scored as a “1” if the individual reported during the interview that a doctor or other healthcare person, like a nurse, had told him/her that he/she *currently* had post-traumatic stress disorder or PTSD.

Other variables included in the analyses were sex, race (African American or Black versus all other races, almost exclusively White), age at the time of the interview, criminal history indicators (age at first arrest, number of prior arrests), and whether the individual reported illegal drug use in the year prior to the current incarceration. Instead of controlling for sex, we stratified the sample by gender. We hypothesized that the survival function might not be the same for men and women following our work with previous prisoner reentry studies (e.g., Lattimore & Visher, 2009, 2021).

Analyses were conducted using Stata 13. For the survival models, goodness of fit statistics indicated that an exponential distribution was the best fit for the data as indicated by the Akaike information criteria (AIC) and Bayesian information criteria (BIC) using the ‘estat ic’ command in Stata. The general recidivism model examined time to first new arrest following release.. The violent recidivism models examined time to first new violent arrest; if the first arrest following release was for a non-violent offense, the observation was censored at the time of first arrest. For the count models, we used negative binomial regression as these models provided the best fit for the count data based on the AIC and BIC.

## Results

The results of our analyses are presented in three subsections. The first section provides an overview of the sample demographics. The second section provides the results from the survival analyses. The third section provides the findings from the negative binomial regression models.

### Descriptive Statistics

The total sample consisted of 564 individuals after listwise deletion of cases with missing data (11 cases or 2%). Table 1 provides information on the variables included in the models. Women were 49% of the total sample. About 59% of the sample identified as African American. Individuals had an average of 11 prior arrests and the average age at first arrest was approximately 20 years. Self-reported prior drug use was high, as 83% reported some drug use in the year preceding their current incarceration. Of the individuals in the sample, 390 (68%) described at least one incident of TBI in their lifetimes and slightly less than 10% reported a current diagnosis of PTSD by a medical professional.

**Table 1:** Sample descriptive statistics

Variable	Frequency	Percent
TBI	390	67.8
PTSD	55	9.6
African American	337	58.6
Female	282	49.0
Prior Drug Use	472	82.5
	**Mean**	**SD**
Time to Violent Arrest	634	332.4
Number of Violent Arrests	0.1	0.4
Time to General Arrest	416	312.6
Number of General Arrests	2	2.3
Age at First Arrest	20	7.0
Total Prior Arrests	11	9.3
Total N	575	100.0

Dependent variables were times to first general (any) and first violent arrests, as well as counts of any and violent arrests experienced following release. As noted, individuals were interviewed between April 2009 and 2010—approximately three months prior to release. Arrest data were obtained in early 2013 and a conservative censor date of December 15, 2012 was used for the analyses to assure time for local law enforcement to submit data for inclusion in the SC SLED files. Thus, the average length of exposure (time at risk of arrest) was 692 days. Approximately 60% of our subjects experienced at least one arrest and 12% experienced at least one violent arrest. The mean time to a general arrest was 416 days and the mean time to a violent arrest for those with a violent arrest was 634 days. Approximately 41% of subjects were arrested more than once during the follow up period. The mean number of arrests was approximately 2 (or 3 for those experiencing at least one arrest post release). The mean number of violent arrests was about 0.1 (or 1.2 for those experiencing at least one).

### Survival analyses

The results from the exponential survival analyses predicting time to general and violent recidivism are presented in Table 2. For general recidivism, neither TBI nor PTSD was a significant predictor of time to recidivism. The statistically significant predictors were those commonly found to be associated with recidivism. Age at first arrest, total number of prior arrests, and self-reported drug use in the year preceding the current incarceration were all associated with increased risk of new arrest. Age was also significantly associated with time to new arrest with older individuals less likely to experience an arrest. A one-year increase in the age of first arrest reduced the instantaneous risk of recidivism by about 3% (hazard rate, HR = 0.97; *p* < 0.01). For each additional prior arrest, the instantaneous risk of recidivism increased approximately 5% (HR = 1.05; *p* < 0.01). Lastly, those who reported drug use in the year prior to their current incarceration were at greater risk of arrest (HR = 1.65; *p* < 0.01). A one-year increase in current age was associated with about a 4% decrease in the instantaneous risk of recidivism (HR = 0.96; *p* < 0.01).

**Table 2:** Exponential survival model results for time to new arrest for general and violent offense

Variables	General Arrest		Violent Arrest	
	Beta Coefficient	Hazard Ratio	Beta Coefficient	Hazard Ratio
TBI	−0.08	0.92	0.60	1.82
PTSD	0.33	1.39	2.13**	8.41**
TBI*PTSD	0.07	1.07	−0.82	0.44
Sex (Stratified)	−0.03	0.97	0.53	1.70
African American	−0.13	0.88	0.22	1.23
Age 1st Arrest	−0.03**	0.97**	− 0.01	0.99
Age	− 0.04**	0.96**	− 0.04*	0.96*
Total Prior Arrests	0.05**	1.05**	0.01	1.01
Total Prior Violent	–	–	0.08	0.98
Drugs In Last Year	0.50**	1.65**	0.35	1.42

* *p* < 0.05

** *p* < 0.01

The results for the violent recidivism survival models are substantially different. The only statistically significant variables are PTSD and age, with the TBI indicator non-significant at the *p* = 0.09 level. Those diagnosed with PTSD were at a substantially increased risk of arrest for a violent crime (HR = 8.41; *p* < 0.01). The results for current age were consistent with the general recidivism findings, with older subjects at less risk of a violent arrest (hazard rate = 0.96; *p* < 0.05). Although slightly over the 0.05 significance threshold, those with TBI experienced about an 82% increase in the instantaneous risk of rearrest for violent offenses. We included an interaction term between TBI and PTSD for both types of recidivism, but the interaction term was not statistically significant in either model.

### Negative binomial regression

Table 3 presents the results from our negative binomial regression models predicting total number of general and violent arrests in the post-incarceration follow-up period. Similar to the results from the survival model, neither TBI, PTSD, or TBI*PTSD is a statistically significant predictor of the number of general arrests. The significant variables are, again, those commonly seen to predict recidivism. African Americans experienced about 24% fewer general arrests than their non-African American counterparts (incident rate ratio, IRR = 0.76; *p* < 0.01). A one- year increase in age at first arrest decreased the number of post-incarceration arrests by about 4% (IRR = 0.96; *p* < 0.01). Similarly, a one- year increase in current age decreased general arrests by about 3% (IRR = 0.97; *p* < 0.01). Lastly, having experienced an additional prior arrest was associated with about a 5% increase in the number of post-incarceration arrests (IRR = 1.05; *p* < 0.01).

**Table 3:** Negative binomial regression results for numbers of new arrests for general and violent offenses

Variables	General Arrests		Violent Arrests	
	Beta Coefficient	IRR	Beta Coefficient	IRR
TBI	−0.14	0.86	0.68*	1.99*
PTSD	−0.13	0.88	1.93*	6.92*
TBI*PTSD	0.27	1.31	−0.93	0.39
Male	0.05	1.05	0.38	1.47
African American	−0.28**	0.76**	0.24	1.27
Age 1st Arrest	−0.04**	0.96**	−0.03	0.97
Age	−0.03**	0.97**	−0.03	0.97
Total Prior Arrests	0.05**	1.05**	0.02	1.01
Total Prior Violent	–	–	0.07	1.07
Drugs In Last Year	0.26	1.30	0.54	1.71

* *p* < 0.05

** *p* < 0.01

TBI and PTSD were the only variables that were significantly associated with the number of post-incarceration violent arrests. Individuals with TBI experienced almost twice (IRR = 1.99; *p* < 0.05) the number of post-incarceration violent arrests as those who reported no TBI. Additionally, individuals reporting a diagnosis of PTSD experienced almost seven times (IRR = 6.92; *p* < 0.05) the number of arrests for violent offenses than individuals not reporting a diagnosis of PTSD. Again, we included an interaction term for both general and violent recidivism between TBI and PTSD, but these interactions were not statistically significant in either model.

## Discussion

This study examined the relationships of TBI and PTSD with two different types of criminal recidivism measured by arrest following release from prison for any (or general) offenses and violent offenses. The results suggest that TBI and especially PTSD are important factors in predicting violent recidivism, but that they do not seem to play a significant role in other forms of criminal behavior. This finding appears to be consistent with findings from recent physiological and neurological work. For example, research has shown that the frontal and temporal lobes are the most commonly affected areas of the brain in those with TBI. Individuals with damage to these areas can experience profound changes in cognitive functioning as well as changes in personality (Cicerone, et al., 2000; Whyte, et al., 1996).

These areas of the brain regulate psychological traits such as self-control and emotional impulses. In a review of this research, Bufkin and Luttrell (2005) found that about 70% of the single-photon emission computed tomography (SPECT) and positron emission tomography (PET) studies they reviewed showed temporal lobe dysfunction in aggressive and violent groups. Others have shown that homicide offenders who had suffered severe child abuse show reduced functioning in the right hemisphere, more specifically, in the right temporal cortex (Raine, 2002).

Interestingly, at least for violent post-release arrest offenses, once both TBI and PTSD are included in the models, many of the well-known predictors of recidivism such as sex, age at first arrest, current age, and total prior arrests are no longer predictive. This has important implications for future research on recidivism and, to a lesser extent, on risk assessment that depends on these traditional indicators. Specifically, it may be that models failing to control for TBI and PTSD may be misspecified, which could lead to biased parameter estimates. It is important for future research to replicate this study in an effort to refine our understanding of the influence of TBI and PTSD on criminal recidivism.

## Conclusions

Although our project fills an important gap in the literature, there are limitations. First, the sample is limited to individuals who were incarcerated in and released from prisons in South Carolina, which may affect the generalizability of the results to correctional populations outside of South Carolina. Second, the presence of TBI relies on retrospective reporting from the individuals in the sample. These individuals may have trouble remembering or recalling TBIs especially if these occurred at an early age. There may also be some concern that incarcerated individuals may self-report TBIs more or less often than those in the general population. However, the validity of self-report of TBI shows similar reliability consistency in the incarcerated population when compared to the general population (Bogner, & Corrigan, 2009; Farrer, & Hedges, 2011; National Center for Injury Prevention and Control, 2003).

Although several studies have found an association between TBI and criminal offending as well as PTSD and criminal offending, most of these studies are descriptive and fail to control for a variety of well-known predictors of crime and recidivism. Additionally, to the extent that these studies are cross sectional or retrospective, it is difficult to establish the causal ordering of the variables. In other words, it is difficult to determine whether PTSD or TBI preceded their criminal offenses.

Our research demonstrates that PTSD and TBI are predictive of future violent offending, as measured by arrest, while controlling for other variables that have been shown to predict recidivism. The results further suggest that the development of appropriate interventions to address TBI and PTSD in prisoner populations may provide an avenue for reducing subsequent violent behavior, as well as the importance of intervention to prevent TBI and PTSD early in life.

## Data Availability

The datasets used and/or analyzed during the current study are available from the corresponding author on reasonable request.

## References

[CR1] Barnfield TV, Leathem JM (1998). Incidence and outcomes of traumatic brain injury and substance abuse in a New Zealand prison population. Brain Injury.

[CR2] Bogner JA, Corrigan JD (2009). Reliability and predictive validity of the Ohio State University TBI identification method with prisoners. The Journal of Head Trauma Rehabilitation.

[CR3] Bufkin JL, Luttrell VR (2005). Neuroimaging studies of aggressive and violent behavior: Current findings and implications for criminology and criminal justice. Trauma Violence Abuse.

[CR4] Cicerone KD, Dahlberg C, Kalmar K, Langenbahn D, Malec J, Bergquist T (2000). Evidence-based cognitive rehabilitation: Recommendations for clinical practice. Archives of Physical Medicine and Rehabilitation.

[CR5] Cohen H, Zohar J, Matar M (2003). The relevance of differential response to trauma in an animal model of posttraumatic stress disorder. Biological Psychiatry.

[CR6] Corrigan JD, Bogner J (2007). Initial reliability and validity of the Ohio State University TBI identification method. The Journal of Head Trauma Rehabilitation.

[CR7] DelBello MP, Soutullo CA, Zimmerman ME, Sax KW, Williams JR, McElroy SL (1999). Traumatic brain injury in individuals convicted of sexual offenses with and without bipolar disorder. Psychiatry Research.

[CR8] Diagnostic and statistical manual of mental disorders (2004). *American Psychiatric Association*.

[CR9] Donley S, Habib L, Jovanovic T, Kamkwalala A, Evces M, Egan G (2012). Civilian PTSD symptoms and risk for involvement in the criminal justice system. The Journal of the American Academy of Psychiatry and the Law.

[CR10] Facer-Irwin E, Blackwood NJ, Bird A, Dickson H, McGlade D, Alves-Costa F, MacManus D (2019). PTSD in prison settings: A systematic review and meta-analysis of comorbid mental disorders and problematic behaviours. PLoS One.

[CR11] Farrer TJ, Hedges DW (2011). Prevalence of traumatic brain injury in incarcerated groups compared to the general population: A meta-analysis. Progress in Neuro-Psychopharmacology & Biological Psychiatry.

[CR12] Faul M, Xu L, Wald MM, Coronado VG (2010). *Traumatic brain injury in the United States: Emergency department visits, hospitalizations, and deaths*.

[CR13] Ferguson P, Pickelsimer E (2011). *Scoring of scales in the statewide investigation of traumatic brain injury in prison (SITBIP) study*.

[CR14] Ferguson PL, Pickelsimer EE, Corrigan JD, Bogner JA, Wald M (2012). Prevalence of traumatic brain injury among prisoners in South Carolina. The Journal of Head Trauma Rehabilitation.

[CR15] Fleminger S, Ponsford J (2005). Long term outcome after traumatic brain injury - more attention needs to be paid to neuropsychiatric functioning. British medical journal.

[CR16] Freeman TW, Roca V (2001). Gun use, attitudes toward violence, and aggression among combat veterans with chronic posttraumatic stress disorder. The Journal of Nervous and Mental Disease.

[CR17] Goff A, Rose E, Rose S, Purves D (2007). Does PTSD occur in sentenced prison populations? A systematic literature review. Criminal Behaviour and Mental Health.

[CR18] Kristiansson M, Sumelius K, Sondergaard HP (2004). Post-traumatic stress disorder in the forensic psychiatric setting. The Journal of the American Academy of Psychiatry and the Law.

[CR19] Kubiak SP (2004). The effects of PTSD on treatment adherence, drug relapse, and criminal recidivism in a sample of incarcerated men and women. Research on Social Work Practice.

[CR20] Langevin R (2006). Sexual offenses and traumatic brain injury. Brain and Cognition.

[CR21] Lattimore, P. K., & Visher, C.A. (2009). Multi-site evaluation of SVORI: Summary and synthesis. Prepared for the National Institute of Justice.

[CR22] Lattimore PK, Visher CA, Lattimore PK, Huebner BM, Taxman FS (2021). Considerations on the multi-site evaluation of the serious and violent offender reentry initiative. *Handbook on moving corrections and sentencing forward: Building on the record*.

[CR23] McFall M, Fontana A, Raskind M, Rosenheck R (1999). Analysis of violent behavior in Vietnam combat veteran psychiatric inpatients with posttraumatic stress disorder. Journal of Traumatic Stress.

[CR24] McFarlane AC (2006). The prevalence and longitudinal course of PTSD. Annals of the New York Academy of Sciences.

[CR25] National Center for Injury Prevention and Control (2003). *Report to congress on mild traumatic brain injury in the United States: Steps to prevent a serious public health problem*.

[CR26] Peltonen K, Ellonen N, Pitkänen J, Aaltonen M, Martikainen P (2020). Trauma and violent offending among adolescents: A birth cohort study. Journal of Epidemiology and Community Health.

[CR27] Pickelsimer EE, Ferguson PL, Cornelius ME, Teklehaimanot A, Johnson ER (2011). *Data codebook of the statewide investigation of traumatic brain injury in prisons study*.

[CR28] Raine A (2002). Annotation: The role of prefrontal deficits, low autonomic arousal, and early health factors in the development of antisocial and aggressive behavior in children. Journal of Child Psychology and Psychiatry.

[CR29] Raine A, Buchsbaum MS, Stanley J, Lottenberg S, Abel L, Stoddard J (1994). Selective reductions in prefrontal glucose-metabolism in murderers. Biological Psychiatry.

[CR30] Ray, B., & Sapp, D. (2014). Traumatic brain injury among Indiana state prisoners. *Journal of Forensic Sciences*, 1–24. 10.1111/1556-4029.12466 24588316

[CR31] Sherman S, Fostick L, Zohar J (2014). Comparison of criminal activity between Israeli veterans with and without PTSD. Depression and Anxiety.

[CR32] Shiroma EJ, Ferguson PL, Pickelsimer EE (2012). Prevalence of traumatic brain injury in an offender population: A meta-analysis. The Journal of Head Trauma Rehabilitation.

[CR33] United States Department of Justice. Office of Justice Programs. Bureau of Justice Statistics. (2013). National Corrections Reporting Program, 2009. ICPSR30799-v2. Ann Arbor, MI: Inter-university Consortium for Political and Social Research [distributor], 10.3886/ICPSR30799.v2.

[CR34] Whyte J, Polansky M, Cavallucci C, Fleming M, Lhulier J, Coslett HB (1996). Inattentive behavior after traumatic brain injury. Journal of the International Neuropsychological Society.

[CR35] Williams WH (2003). Brain injury and emotion: An overview to a special issue on biopsychosocial approaches in neurorehabilitation. Neuropsychological Rehabilitation.

[CR36] Williams WH, Cordan G, Mewse AJ, Tonks J, Burgess CNW (2010). Self-reported traumatic brain injury in male young offenders: A risk factor for re-offending, poor mental health and violence?. Neuropsychological Rehabilitation.

[CR37] Williams WH, Mewse AJ, Tonks J, Mills S, Burgess CN, Cordan G (2010). Traumatic brain injury in a prison population: Prevalence and risk for re-offending. Brain Injury.

